# Underwater Wireless Sensor Communications in the 2.4 GHz ISM Frequency Band

**DOI:** 10.3390/s120404237

**Published:** 2012-03-28

**Authors:** Jaime Lloret, Sandra Sendra, Miguel Ardid, Joel J. P. C. Rodrigues

**Affiliations:** 1 Integrated Management Coastal Research Institute, Universidad Politécnica de Valencia, C/Paranimf, n° 1, Grao de Gandia 46730, Spain; E-Mails: sansenco@posgrado.upv.es (S.S.); mardid@fis.upv.es (M.A.); 2 Instituto de Telecomunicações, Universidade da Beira Interior, Rua Marquês d'Ávila e Bolama 6201-001 Covilhã, Portugal; E-Mail: joeljr@ieee.org

**Keywords:** Underwater Wireless Sensor Network (UWSN), underwater communication, 2.4 GHz, high data rates, electromagnetic waves

## Abstract

One of the main problems in underwater communications is the low data rate available due to the use of low frequencies. Moreover, there are many problems inherent to the medium such as reflections, refraction, energy dispersion, *etc.*, that greatly degrade communication between devices. In some cases, wireless sensors must be placed quite close to each other in order to take more accurate measurements from the water while having high communication bandwidth. In these cases, while most researchers focus their efforts on increasing the data rate for low frequencies, we propose the use of the 2.4 GHz ISM frequency band in these special cases. In this paper, we show our wireless sensor node deployment and its performance obtained from a real scenario and measures taken for different frequencies, modulations and data transfer rates. The performed tests show the maximum distance between sensors, the number of lost packets and the average round trip time. Based on our measurements, we provide some experimental models of underwater communication in fresh water using EM waves in the 2.4 GHz ISM frequency band. Finally, we compare our communication system proposal with the existing systems. Although our proposal provides short communication distances, it provides high data transfer rates. It can be used for precision monitoring in applications such as contaminated ecosystems or for device communicate at high depth.

## 1. Introduction

Nowadays, there is extensive ongoing research activity relating to underwater communications and underwater sensor networks. On one hand, the main research lines are based on increasing the distance and bandwidth, and, on the other hand, the attempt to reduce the energy consumption of underwater devices, with the aim of increasing the network lifetime [1,2]. Underwater communication research is primarily focused on the use of optical signals, electromagnetic signals and the propagation of acoustic and ultrasonic signals. Each technique has its own characteristics, with its benefits and drawbacks, mainly due to the chemical characteristics [3] and physical constraints of the medium [4,5].

Systems based on optical communication are able to reach very high propagation speeds. However a strong backscattering is caused by suspended particles and they are affected by the turbidity of the water, so they are not good options for long distances.

Systems based on acoustic waves are less sensitive to fine particles suspended in the water and to the water turbidity, than the optical waves. Moreover, they are the most used methods, since they are able to reach large distances (over 20 km [6]). Although acoustic communication is a proven technology, it presents some main drawbacks, like the low data rate (0 b/s to 20 kb/s), which is limited by some factors, such as low carrier frequency, strong reflections and attenuation when the communication is performed near the surface, as well as poor performance in turbid water with large particles, sensitivity to varying environmental characteristics and the salinity. In acoustic and ultrasonic communications, researchers usually work on varying the type of modulation and communication protocol, in order to minimize the effects of reflections, and on achieving as high a communication data rate as possible.

When higher data rates are needed, we should make use of radio frequency (RF) methods, which are able to reach communication data rates of up to 100 Mb/s in very short distances, apart from presenting substantial immunity from the environmental features. Electromagnetic (EM) waves, in the RF range, can also be a good option for underwater wireless communication systems. EM waves are less sensitive to reflection and refraction effects in shallow water than acoustic waves. In addition, suspended particles have very little effect on them. The speed of EM waves is higher (150,000 times greater) than that of acoustic ones The speed of an EM wave mainly depends on permeability (*μ*), permittivity (*ε*), conductivity (*σ*) and volume charge density (*ρ*) [7]. These parameters change with the type of water and the electrical conductivity value associated with the medium often varies, thus the wave propagation speed and absorption coefficient, which are directly related to the working frequency, also vary. Conductivity presents different values for each case, seawater has a high conductivity average value, which is around 4 S/m (obviously it changes with the salinity and physical properties of each kind of sea water), but in fresh water the typical value is 0.01 S/m and drinking water presents a conductivity between 0.005 and 0.05 S/m. Moreover, the permittivity of seawater changes as a function of the frequency, the temperature and the salinity. In [8,9], authors provided a relationship model of this dependency in the water. Thus, the main problem for underwater communications based on EM waves is the high attenuation due to the conductivity of the water. This attenuation increases when the EM wave frequency increases. Hence, the higher frequencies will register greater signal losses.

In this paper, we perform a practical study of the behavior of EM signals (in the 2.4 GHz ISM frequency band) in underwater environments, using devices compatible with the IEEE 802.11 standard [10]. We have analyzed other technologies that also work on this frequency. This is the IEEE 802.15.4 standard [11]. *A priori*, we think that, due to the low-power consumption of IEEE 802.15.4, it would be better to use these devices as sensor nodes. However, our application needs data transfer rates higher than the ones offered by IEEE 802.14.5. For this reason, we should sacrifice a little power consumption in favor of improved data transfer rates. Table 1 shows a comparison of the maximum data transfer rates of both wireless technologies.

The paper shows the tests performed at different frequencies and modulations in order to measure several parameters such as minimum depth, distance between devices and signal transmission characteristics. These tests were performed in a swimming pool filled with fresh water. We set up an underwater point-to-point link between two sensor nodes. These underwater sensor nodes were developed by us. We used two computers connected to each sensor node via serial in order to monitor the activity of the underwater point-to-point link between sensors. We have used the echo request and echo reply packets in order to perform our tests.

From the point of view of applications, it is easy to think that underwater communication in the 2.4 GHz band is unhelpful and impractical because water has a high attenuation of these frequencies. However, as we shall see at the end of this paper, there are many applications where the use of EM waves brings many benefits.

The rest of the paper is structured as follows: Section 2 reviews some published works on underwater wireless transmission based on RF and acoustic communications. Section 3 summarizes the main issues to be considered in underwater communication in fresh water when electromagnetic waves are used. It also shows the most important features of each modulation used in our research. The fourth section shows the deployed sensor node and its consumption. The used topology and the measurement strategies are also explained in this section. Section 5 shows the results obtained as a function of the working frequency and the distance between devices. The analytical models obtained from the real measurements are shown in Section 6. In Section 7, we compare our 2.4 GHz communication system proposal with the communication proposals published in the related literature. Finally, Section 8 contains the conclusions and future work proposals.

## 2. Related Work

Many underwater communication deployments use acoustic or low frequency technologies, which is why the number of works in higher frequencies is very scarce. We have found some papers showing comparative studies regarding the transmission characteristics of the acoustic, optical and electromagnetic signals in underwater environments. There is a huge variety of articles describing the propagation of acoustic waves. An example of a path loss analysis given by the reflection and refraction of the waves is provided in [12]. Moreover, we can see in [13] the effects of depth and temperature in this type of wave. We can also find a variety of studies about the propagation and losses in optical communications [14].

There is very little literature published about EM waves because this technology is not used in underwater communications. There is not too much documentation about high frequency in underwater communications because most of the works are designed for low frequencies in order to achieve large communication distances, preventing the power losses generated in high frequencies.

Chakraborty *et al.* presented a detailed description of the relationship between several propagation parameters of electromagnetic waves [6]. They studied skin depth, propagation velocity, total path loss, wavelength and frequency for different values of distance and conductivity of the water medium for underwater communication. They confirmed that EM wave propagation is characterized mainly by four parameters: permeability, permittivity, conductivity and volume charge density.

In RF communications, researchers work with Very Low Frequency (VLF), decreasing the frequency in order to have a more effective range of communication. Concretely, some researchers of the Swansea Metropolitan University, U.K., performed their simulations at 3 KHz and distances between nodes of about 40 meters [15].

In [16], Frater *et al.* compared RF and acoustic communications. They measured the maximum distances for RF. The paper shows the maximum distances for several frequencies (approximately 6 m at 100 kHz, 16 m at 10 kHz, and 22 m at 1 kHz). They concluded that RF communication offers higher performance than acoustic communication in certain ranges.

Anguita *et al.* dismissed the RF method for underwater communication because they reported that it is strongly attenuated [17]. Thus they took as invalid the 2.4 GHz frequency. However EM signals offer higher throughputs than acoustic signals by up to an order of magnitude. For example, Nowsheen *et al.* [18,19] developed an FPGA-based modem, that used frequencies ranging from 100 kHz to 1 MHz and BPSK modulation, with shipments of data packets with a duration of 1 ms with a wait time of 20 ms. They stated that it is an appropriate interval to avoid the effects of reflections in the tank.

In [20], Jiang and Georgakopoulos conducted a study of the EM wave propagation in fresh water for frequencies between 23 kHz and 1 GHz. This work presents two analyses on electromagnetic waves. On one hand, the authors measured the transmission loss given by the reflection at the air-water interface and, on the other hand, they analyzed the propagation loss inside the water due to its physical properties. They conclude that the propagation loss increases slowly for frequencies up to 1 MHz, while it remains constant between 1 MHz to 100 MHz and then increases dramatically for higher frequencies. In addition, the total loss in the 3–100 MHz frequency range, for a depth of 1 m, is from 10 dB to 45 dB smaller than the loss in lower and higher frequencies. Finally, they tested a half-wavelength loop antenna operating at 100 MHz inside fresh water and they concluded that a diameter of only 5.3 cm is needed for the antenna.

In [21], the authors of this paper performed a study on RF communication in the 2.4 GHz ISM frequency band. We measured the number of lost packets and round trip time for 1, 2, 5.5 and 11 Mbps at different frequencies and different modulations for different distances between underwater wireless sensors. Except for the last paper presented in this section, which is our paper, we have not found any other papers in the related literature showing the performance of underwater communication tests at 2.4 GHz.

## 3. Impact of the Environment on the EM Waves and Transmission Techniques

This section shows the main expressions followed by the EM waves in the underwater environment and the factors that may cause drawbacks in the communication operation. We also see the main modulations that are used in IEEE 802.11 standard.

### 3.1. Experimental Expressions for Underwater Communications in Fresh Water

EM waves have several advantages over acoustic waves when used to transmit signals in water. They provide fast and efficient communication between network nodes, and, because they use higher work frequencies, they provide higher data rates. There are several factors that limit the use of EM waves in the water. EM waves are propagated in very different ways depending on the type of water where the communication system is implemented.

Freshwater is a medium that has low loss. The propagation speed of the signals *c* can be expressed by following the approximation shown in Equation (1) [22]:

(1) $$\text{c}\approx \frac{1}{\sqrt{\left(1+\chi_{e}\right)\cdot 8.85\cdot 10^{-12}\cdot \mu_{r}\cdot 4\pi \cdot 10^{-7}}}$$

where *X*_e_ the electric susceptibility of the medium and *μ_r_* is the magnetic permeability of medium (in this case, it is the water).

The absorption coefficient *α* for the propagation of EM in freshwater can be approximated by Equation (2) [22]:

(2) $$\alpha \approx \frac{\sigma }{2}\sqrt{\frac{\mu_{r}\cdot 4\pi \cdot 10^{-7}}{\left(1+\chi_{e}\right)\cdot 8.85\cdot 10^{-12}}}$$

where σ is the electrical conductivity, *X*_e_ the electric susceptibility of the medium and *μ_r_* is the magnetic permeability of medium (in this case, it is the water). Equations (1) and (2) show that the wave propagation and absorption coefficients in freshwater are independent of the working frequency of the transmitted signals.

### 3.2. Modulations

Modulating the signal means applying techniques that modify the signal in order to facilitate the transmission of information through the communication channel. Finally, the signal should be demodulated to its original form at the other end. The transmitted signal is called modulated signal.

Different types of modulation can be used: pulse modulation and continuous wave modulation. In addition, the carrier signal can be analog or digital. In our experiments, continuous-wave modulation is used. Specifically, we used the phase shift keying modulation (PSK) [23] and Complementary Code Keying modulation (CCK) [24].

In the PSK modulation, the phase of the carrier varies, while the amplitude of the carrier remains constant. For this reason, the phase has discontinuities that appear at the beginning and at the end of each symbol interval *T*. We can distinguish two alternatives:

- *Conventional PSK*. The phase of a given symbol or state is referred to the phase of the unmodulated carrier.
- *Differential PSK*. The phase of a given symbol or state is referred to the phase of the previous state.

Both alternatives have several modulation subtypes. They depend on the number of symbols of phase displacement. The most common ones are BPSK (Binary PSK), QPSK (Quaternary PSK), 8PSK, 16PSK, OQPSK (Offset Quaternary PSK) and SOQPSK (Shaped OQPSK), where the main difference is the amount of output phases for a single carrier frequency that each modulation presents.

### 3.3. Modulations in the IEEE 802.11b/g Standard

Because our tests were performed using commercial devices, operating under IEEE 802.11b/g, we discuss the standard and identify each type of modulation with the data rates specified in the standard [11].

The IEEE 802.11 standard defines the use of different modulation types, depending on the transmission speed. The choice is made depending on the application. BPSK and QPSK modulations are optimal from the point of view of error protection, but BPSK is used in low-cost transmitters that do not require high speeds. CCK modulation allows encoding multiple bits of data directly on a single chip with eight 64-bit sequences. Therefore, the CCK method can achieve a maximum speed of 5.5 Mbps, by encoding 4 bits of data at a time, or up to 11 Mbps by encoding 8 bits of data, in the band of 2.400 GHz to 2.4835 GHz. The wireless local area networks operating under the IEEE 802.11b and IEEE 802.11g variants, allows a variety of modulations. Wireless networks based on IEEE 802.11g standard employ CCK when operating at IEEE 802.11b speeds. At higher speeds (up to a theoretical maximum of 54 Mbps), IEEE 802.11g uses a transmission scheme called orthogonal frequency-division multiplexing (OFDM).

## 4. Sensor Node Description and Test Bench

In order to carry out our test, we developed two underwater wireless sensor nodes. The test performance was performed in a swimming pool. In this section, we describe the developed wireless sensor node and explain the hardware and software used for our tests.

### 4.1. Wireless Sensor Node Description

A wireless sensor node is an electronic device that is used as an interface between the physical parameters, which can be detected within a medium, and a wireless data network [25,26]. When they are deployed in the seawater, they have some common specific parameters [9]. A node is made up of four main parts: (1) a power unit, consisting of a battery and a number of DC/DC converters; (2) a processing unit, which usually consists of a small processor and memory; (3) the physical sensors and (4) the transceiver circuit that is formed by a transmitter and a receiver system.

**Figure 1.**
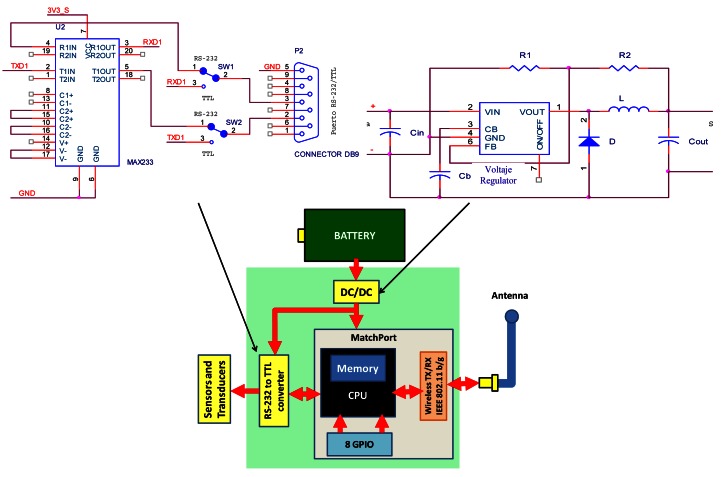
Block diagram of the underwater wireless sensor node.

In order to provide a wireless interface card to the sensor node we used the MatchPort b/g [27], from Lantronix^®^, Inc. It is an embedded system that acts as a gateway between a wireless network, based on IEEE 802.11b/g standard, and a 10/100 Ethernet-based wired data network. Two sets of pins are incorporated to implement two transistor–transistor logic (TTL) ports (but can be converted to RS-232 or RS-485 interfaces) and eight GPIO (which are configurable from its graphical interface) that allow controlling sensors based on ON/OFF operation systems. The device uses CMOS technology with 3.3 V logic levels. The operating speeds range from 300 bps to 921 Kbps. The frames can be 7 or 8 bits with 1–2 stop bits. They can also be configured with even/odd parity, or no parity, and we can use flow control, using the signals (CTS/RTS) or not, and simply use the TX and RX signals. Matchport works with System-on-Chip (SoC) processor with 256 KB SRAM, 2 MB Flash memory for storing web pages and the device firmware. MAX233CPP integrated circuit converts the signals from the MatchPort from TTL levels to RS-232 standard logic level signals. We have used this integrate circuit, because it requires less passive components than others. Other models need several resistors to limit the current flow of its entries. This current limitation procedure causes higher power consumption. Therefore, a simpler circuit with the same TX/RX features means lower power consumption. The sensor node has lower power consumption using this configuration than using other configurations. In order to power the device with batteries, we used a LDO voltage regulator and a small capacitor 98AGL52B [28] to filter the output voltage and prevent voltage fluctuations. Figure 1 shows the block diagram of the circuit.

We can see that the MatchPort includes the main elements of a sensor node, such as CPU, memory and radio system. We can also see the schematic for TTL/RS232 converter, which communicates the node with the sensors via DB9 connector, and DC/DC circuit which transform the voltage of 12 volts to 3.3 volts.

**Figure 2.**
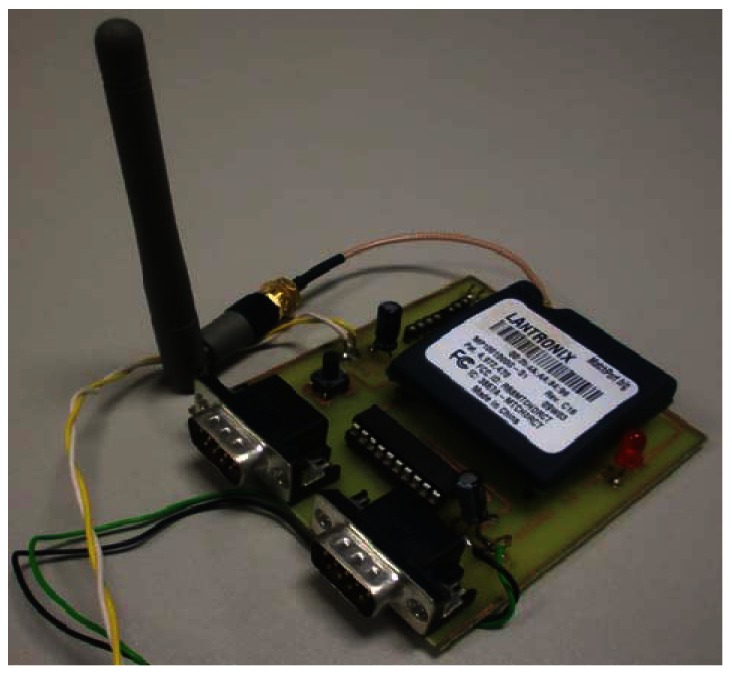
Image of the underwater wireless sensor node.

In our underwater wireless sensor node, the MatchPort acts as a central processing unit and transmitting device. The device allows us to connect 2 sensors with RS-232, TTL or RS-485 interfaces, which are one of the most common interfaces in underwater sensors. Its frequency range is from 2.412 GHz to 2.472 GHz. These values correspond to the spectrum used by devices operating under the IEEE 802.11b/g standard at 2.4 GHz. The used antenna is a monopole with 2 dBi of gain. Figure 2 shows the model in its first phase of development to perform our tests.

### 4.2. Scenario

**Figure 3.**
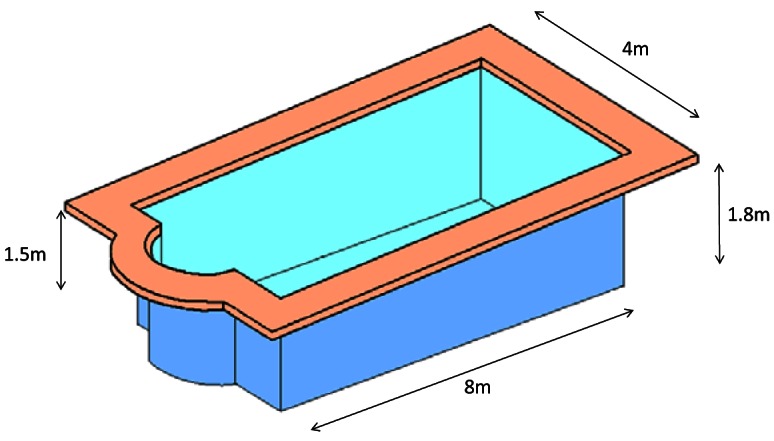
Swimming pool used to take measurements.

In order to take measurements, we used a swimming pool which has 32 m^2^ surface with a length of 8 meters and 4 meters wide. It is built with brick walls that are covered with small mosaic tiles. The swimming pool depth ranges between 1.5 m and 1.80 m. We chose this position in order to avoid any reflection on the walls, ground and surface water due to the change of medium. Reflections are avoided because the measured distances have a lower order of magnitude than the dimensions of the pool. Figure 3 shows the sketch of the swimming pool used to gather the measurements.

Measurements were taken in fresh water. It had a temperature of 26 °C. In addition, the pH value was 7.2 and the amount of chlorine and bromine dissolved in the water was 0.3 mg/L.

### 4.3. Sensor Node Preparation

In order to perform the tests, we placed the device in a sealed plastic box to make it both watertight and airtight. This also allowed communication with other devices in the network. To maintain the upright position of the device, we estimated the required ballast. It was the container volume in liters and an additional weight equal to the half value of the calculated volume.

**Figure 4.**
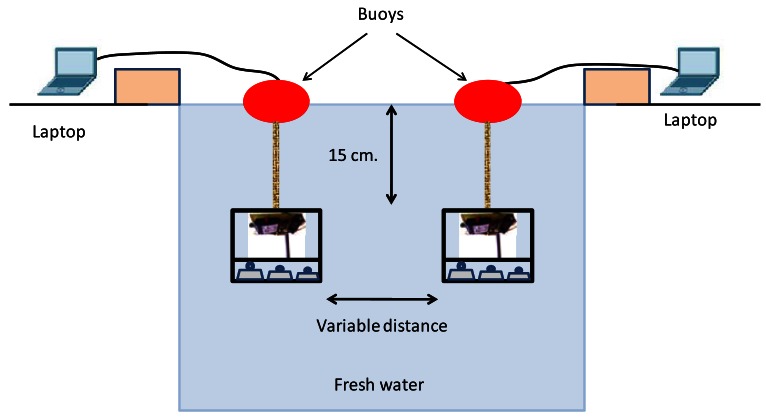
Topology used to take measurements.

Our first step was to ensure that all measurements taken were valid, thus we had to check that the signal did not spread out of the water. Hence, we determined the minimum depth where the antennae should be placed. In order to do this, we established an ad hoc wireless connection between the node and a laptop outside the water. The wireless sensor node was introduced inside the water and we immersed it until the laptop placed outside did not receive any signal from the wireless sensor node. We lost the wireless signal when it was at 15 cm deep. This simple test ensures that there is no signal gathered from outside. The system is located in the center of the pool, about 1.8 m from the edge of the pool, to avoid any effect of reflections. To carry out the wireless communication tests, we used two wireless sensor nodes under the water. We also used two laptops, located outside the water, connected to each node via serial cable, to gather the data and monitor the network activity. Both antennae were oriented to the bottom with their radiation pattern to down. The gain of both antennae was 2 dBi. The antenna consisted of a single radiating arm vertically straight. This antenna is completed by a ground plane to operate properly. This ground plane can be natural (a water surface to facilitate electron conduction) or artificial (a number of drivers which are joined at the base of the monopole). Hence, both sensors have identical features. Figure 4 shows the topology used to take the measurements.

## 5. Performance Results

**Figure 5.**
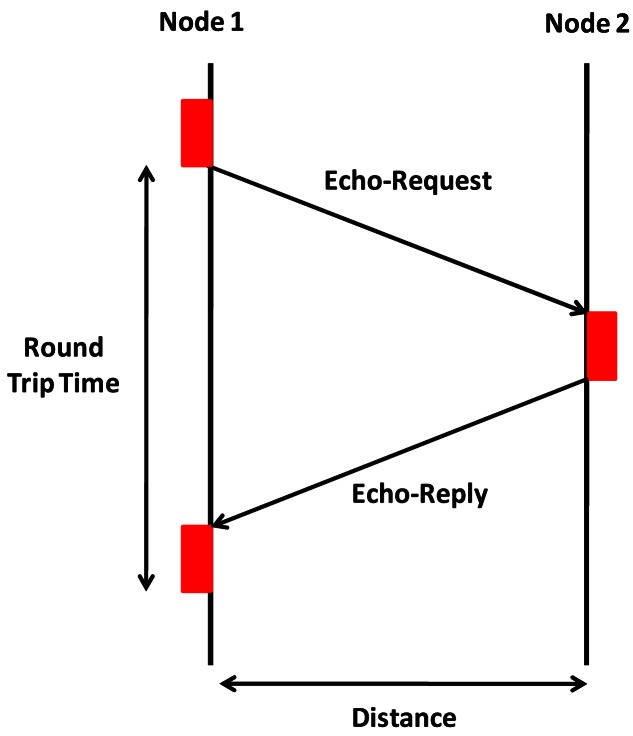
Packet flow diagram.

In order to analyze the performance of our system, we carried out different tests in the 2.4 GHz frequency band with different modulations and transfer rates, while we varied the distance between the antennae. These tests allow us to measure the performance of the developed nodes and characterize its behavior in terms of number of lost packets, round trip time (RTT), modulation techniques for underwater transmission, and the maximum data transfer rates that can be obtained for each modulation. We have used some common commands in the command-line shell interface that let us check the status of the network connection. Concretely, we have used the echo request and echo reply packets in order to perform our tests (see Figure 5). We sent a continuous packet flow and we collected the results. The system performance was evaluated in terms of consumption of sensor node, communication distance, data transfer rate, average RTT and % of lost packets for each frequency.

The calculation of the average RTT has been done, taking into account of only the packets that performed the round-trip successfully. When a packet was not received or was received wrong, we assign the value of 3,000 ms to draw it in the graph, but this value is not taken into account in the average RTT estimation for that case. We have used a threshold value of 3,000 ms, because it is commonly used [29].

Tests have been performed in the first seven channels specified of the 2.4 GHz frequency band in the IEEE 802.11b/g standard. These frequencies correspond to 2.412 GHz, 2.417 GHz, 2.422 GHz, 2.427 GHz, 2.432 GHz, 2.437 GHz and 2.442 GHz. We only tested these frequencies because after the seventh channel we found that the value of lost packets is around 90–100%, which is a very high value for a communications system. Table 2 shows the modulations and data rates used in our performance tests.

We did not include the OFDM transmission scheme in our test performance because when we used this transmission scheme, we obtained even worse measurements and, thus worse behavior than for the other three modulations shown in Table 2.

Because one of the requirements to be met by a wireless sensor node is to have low power consumption in order to prolong the network lifetime [30], our first step has been to measure this consumption. Then, for each modulation and data rate shown in Table 2, we measured the RTT the amount of lost packets between both wireless sensor nodes, while varying the distance between the antennae and the working frequencies in the 2.4 GHz ISM frequency band. It let us know the wireless communication performance and the communication behavior at these frequencies. Hence, we measured the behavior of the modulation BPSK, QPSK and CCK with transfer rates up to 1 Mbps, 2 Mbps, 5.5 Mbps and 11 Mbps. Each test was 3 minutes long. We assigned the value of 3,000 ms to those packets which were not received or were received wrong. From this value, we know that no echo will be received. We know this, due to the wave propagation speed through water and the distance between transmitter and receiver.

### 5.1. Power Consumption

**Figure 6.**
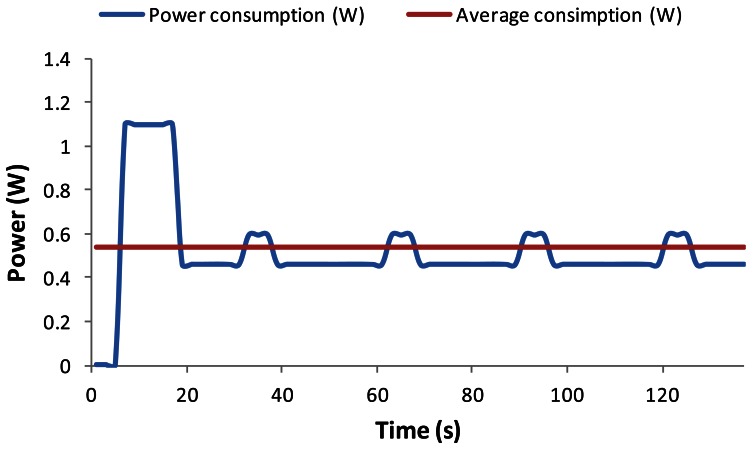
Underwater wireless sensor node consumption.

The wireless sensor node is powered with 3.3 V, with average power consumption in active mode of 460 mW. We observed that when the device is transmitting or receiving data, the power consumption increases to 594 mW, while the device consumes around the 1.1 W in its initialization phase (which is approximately 10 s long). The behavior of the device was monitored for 2.5 min since it was started. After this time, it sent broadcasts every 30 s. Figure 6 shows the energy consumption evolution and the average power consumption of this device during that time.

### 5.2. Performance of BPSK Modulation with 1 Mbps Data Transfer Rate

**Figure 7.**
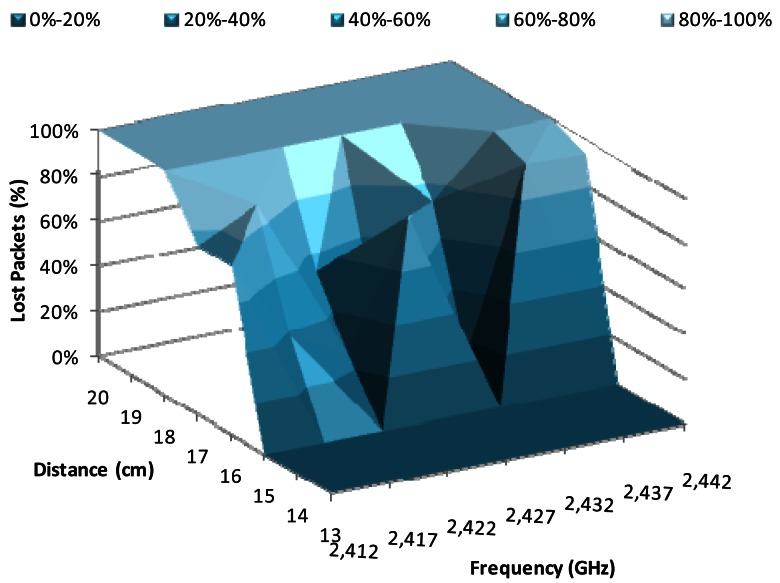
Lost packets for 1 Mbps.

Figure 7 shows the percentage of lost packets when there is a data transmission rate of 1 Mbps, using BPSK modulation, as a function of the working frequency and the distance between devices. The major changes were produced from 15 cm to 18 cm, where the percentage of lost packets is 0% at 15 cm and 100% at 18 cm. We observe that the frequencies with lowest lost packet values were 2.422 GHz and 2.432 GHz, for a distance of 16 cm, while the lowest losses for 17 cm were registered at 2.427 GHz.

**Figure 8.**
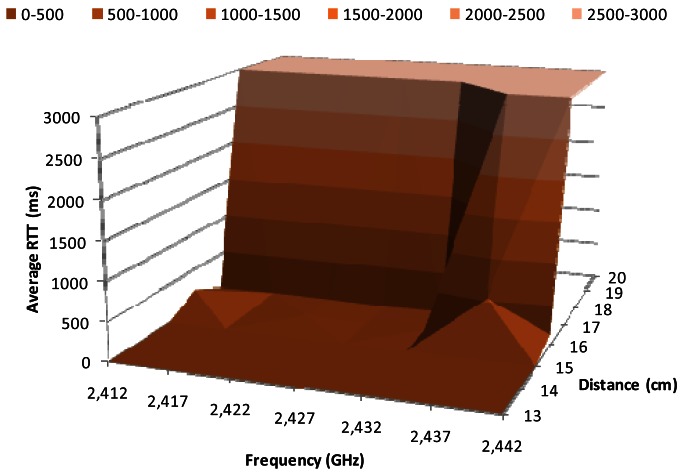
Average RTT for 1 Mbps.

Figure 8 shows the average RTT in milliseconds for 1 Mbps data transfer rate, when the BPSK modulation is used, as a function of the working frequency and the distance between the wireless sensor nodes. We observe that the highest variations occur between 15 cm and 18 cm. The RRT value for 15 cm is close to 3 ms, while it is 3,000 ms for 18 cm. The average RTT value for distances between 15 cm and 18 cm (at 2.412 GHz, 2.417 GHz, 2.422 GHz, 2.427 GHz and 2.432 GHz) is relatively small, around 20 ms. But at 2.437 GHz the RTT value for 16 cm increases up to 500 ms, while for 17 cm there are not registered packets, thus the obtained RTT is 3,000 ms.

### 5.3. Performance of QPSK Modulation with 2 Mbps Data Transfer Rate

**Figure 9.**
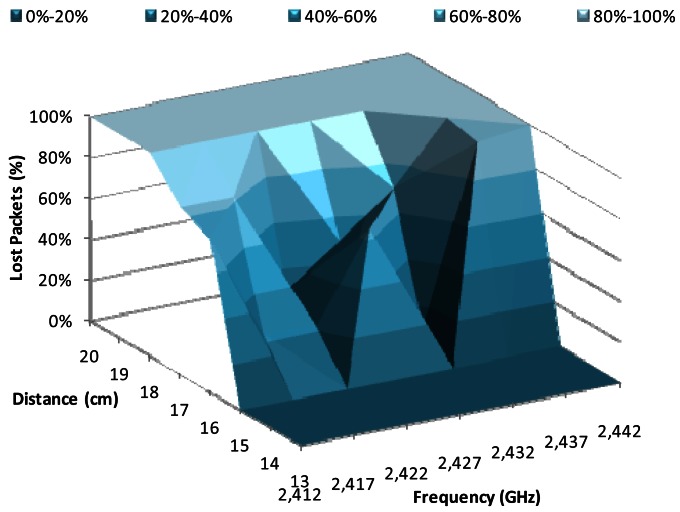
Lost packets for 2 Mbps.

Figure 9 shows percentage of lost packets for 2 Mbps data transfer rate, using the QPSK modulation, as a function of the working frequency and the distance between devices. The percentage of lost packets for 15 cm is around 0%, whereas the number of lost packets is very close to 100% at 18 cm. We observed higher variations for distances between 15 cm and 18 cm. We observe that the frequencies with lowest lost packets percentage are 2.417 GHz, 2.422 GHz and 2.432 GHz, for a distance of 16 cm, while for 17 cm the lowest losses are given at 2.422 GHz.

**Figure 10.**
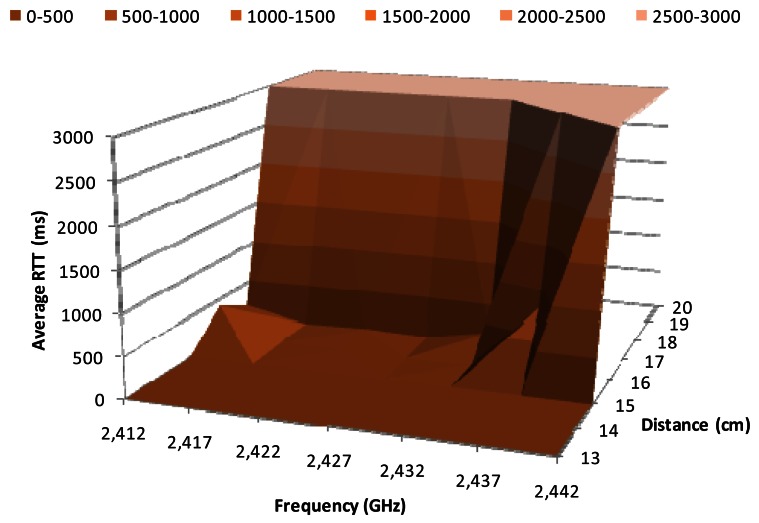
Average RTT for 2 Mbps.

Figure 10 shows the average RTT, in milliseconds, for 2 Mbps data transfer rate, when QPSK modulation is used, as a function of the working frequency work and the distance between wireless sensor nodes. The average RTT values for distances between 15 cm and 18 cm are kept below 500 ms for a frequency of 2.432 GHz, while at 2.437 GHz the average RTT increases up to 1,000 ms when there is a distance of 16 cm, and up to 3,000 ms when there is a distance of 17 cm. We observe RTT average values around 3 ms for distances below 15 cm, and for distances above 18 cm we obtained 3,000 ms.

### 5.4. Performance of CCK Modulation with 5.5 Mbps Data Transfer Rate

**Figure 11.**
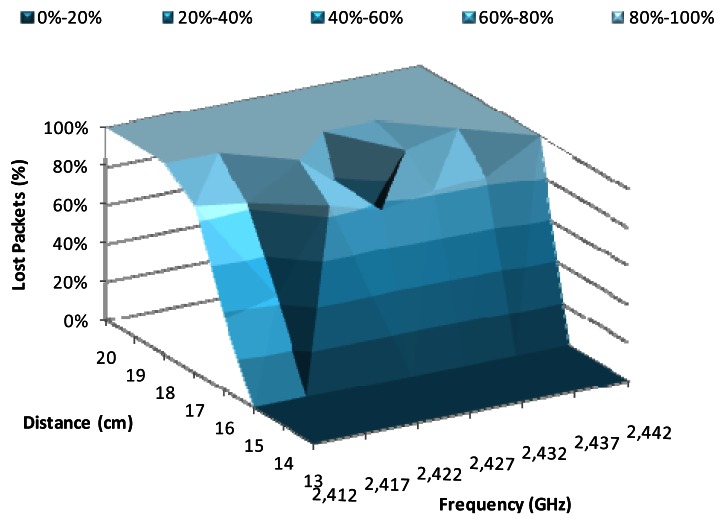
Lost packets for 5.5 Mbps.

Figure 11 shows the percentage of lost packets for 5.5 Mbps data transfer rates, using CCK modulation, as a function of the working frequency and the distance between devices. In this case we observed that the frequencies with the lowest percentage of lost packets are 2.417 GHz, 2.422 GHz and 2.432 GHz, for a distance of 16 cm, while for 17 cm the lowest percentage of lost packets are given at 2.422 GHz. We can see that the percentage of lost packets has increased almost threefold at 2.412 GHz and 2.417 GHz for 16 cm and 17 cm respectively, compared with the other frequencies. Moreover, for 17 cm, the frequency that registers the lowest lost packets percentage is 2.427 GHz, while for 16 cm, only 2.412 GHz and 2.417 GHz had losses below 50%.

**Figure 12.**
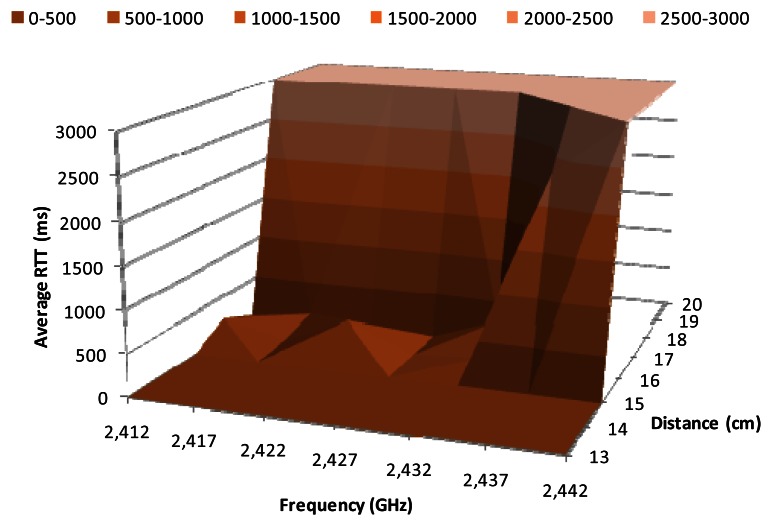
Average RTT for 5.5 Mbps.

Figure 12 shows the average RTT in milliseconds for 5.5 Mbps data transfer rate, when CCK modulation is used, as a function of the working frequency and the distance between devices. In this case, the RTT values for distances of 16 cm and 17 cm are kept below 500 ms for 2.432 GHz, while at 2.437 GHz the RTT value increases up to 2,000 ms for 16 cm, and up to 3,000 ms for 17 cm. The biggest RTT variations are observed for distances between 15 and 18 cm, the measurements obtained beyond this range remain quite stable, between 3 ms and 4 ms for distances below 15 cm and more than 3,000 ms for distances above 18 cm.

### 5.5. Performance of CCK Modulation with 11 Mbps Data Transfer Rate

**Figure 13.**
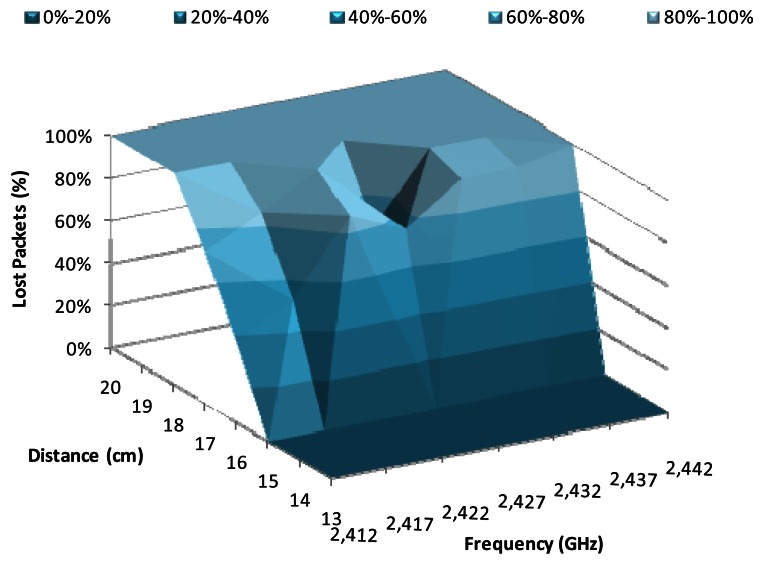
Lost packets for 11 Mbps.

Figure 13 shows the percentage of lost packets for 11 Mbps data transfer rate, when CCK modulation is used, as a function of the working frequency and the distance between wireless sensor nodes. The highest variations have also been observed for distances between 15 and 18 cm where their values are around 0–1% of lost packets for 15 cm and 100% of lost packets for 18 cm and larger distances. We observed that the percentage of lost packets for 16 cm increases almost linearly with the working frequency, and 2.412 GHz and 2.417 GHz have a lost packets percentage below 70%, while for distances of 17 cm, the lost packet percentage values are always above 70% (except for 2.427 GHz).

**Figure 14.**
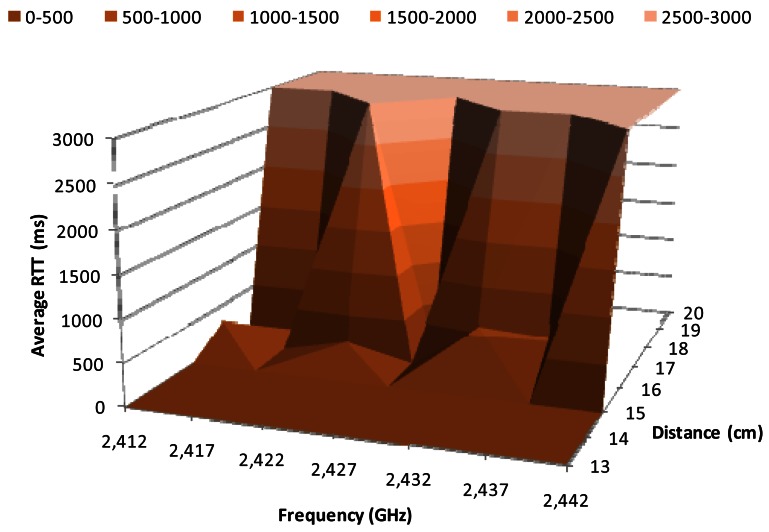
Average RTT for 11 Mbps.

Figure 14 shows the average RTT in milliseconds for 11 Mbps data transfer rate, using CCK modulation, as a function of the working frequency and the distance between devices. The average RTT values obtained for 16 cm remain between 400 ms and 600 ms at frequencies below 2.437 GHz, while at 2.442 GHz we did not measured the RTT of any packet. In 17 cm, the average RTT values are very low for 2.412 GHz, 2.417 GHz and 2.427 GHz, but at other frequencies it reached 3,000 ms. We obtained the same behavior than in the previous cases for distances below 15 cm and for distances above 18 cm.

### 5.6. Summary

Table 3 shows a summary with the best results for each case of the measurements previously shown. It specifies the frequencies with lowest lost packet values and their average RTT value in milliseconds. We have specified the best case for each distance between wireless sensor nodes (from 15 cm to 18 cm), because the biggest variations are registered between these distances.

## 6. Experimental Models of Underwater Communication Using EM Waves in the 2.4 GHz ISM Frequency Band in Fresh Water

After having analyzed the performance of an underwater communication using EM waves depending on the working frequency and the distance between the wireless sensor nodes, in this section we analyze this behavior graphically for each type of modulation. Gathered measurements let us estimate the analytical expressions followed by the underwater communication in each case. Initially we estimated the average RTT value and the average percentage of lost packets for all tested frequencies. In this section, we graphically compare the behavior of the average RTT and the average percentage of lost packets with our models and we will provide the sixth degree polynomial expressions they follow.

### 6.1. Analytical Study for 1 Mbps Data Transfer Rate

**Figure 15.**
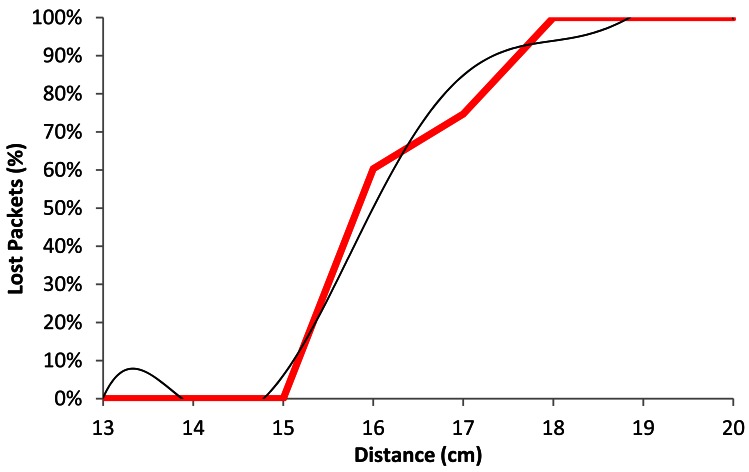
Average lost packets for 1 Mbps.

Figure 15 shows the comparison of the average percentage of lost packets (red line) with our model (black line) as a function of the distance, when the wireless sensor nodes are using BPSK modulation with a data rate of 1 Mbps. We observe that for distances shorter than 15.5 cm, the number of lost packets does not exceed 20%. The percentage of lost packets increases drastically up to 70% at 16.5 cm. There are between 90 and 100% of lost packets at 18 cm.

The black line shown in Figure 15 follows a polynomial expression of degree 6 (shown in Equation (3)). The correlation coefficient between both curves (*R^2^*) is 0.981:

(3) $$\begin{array}{c} \text{Lost}\_{\text{Packets}}_{1\text{Mbps}}\left(\%\right)=\left|-0.001x^{6}+0.121x^{5}-5.019x^{4}+110.2x^{3}-1.353x^{2}+8.817x-\;23.796\right|\\ R^{2}=0.981 \end{array}\;$$

where *Lost_Packets*_1_*_Mbps_*(%) is the percentage of lost packets for 1 Mbps data transfer rate and *x* is the distance between devices, in cm.

**Figure 16.**
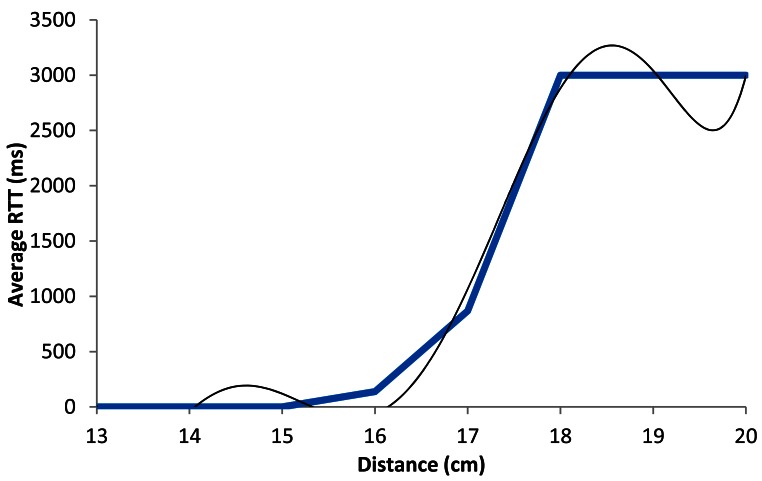
Average RTT for 1 Mbps.

Figure 16 shows the graph of the average RTT in ms as a function of the distance when the devices are working using BPSK modulation with 1 Mbps data transmission rate. The blue line shows the estimated average of the measured packets. The black line shows our estimated model. We can see that for lower distances than 16.5 cm, the RTT value is always lower than 500 ms, but when distance increases, the average RTT value increases up to 1,000 ms (when there is a distance of around 17 cm). At 18 cm this value is approximately arrives to 3,000 ms because there are many lost packets.

The black line shown in Figure 16 follows a degree 6 polynomial expression. It is shown in Equation (4) The correlation coefficient (*R^2^*) between both lines is 0.992:

(4) $$\begin{array}{c} {\text{RTT}}_{\text{Aver}\_1\text{Mbps}}\left(\text{ms}\right)=\left|6.926x^{6}-681.2x^{5}+27.772x^{4}-60.071x^{3}+7\cdot 10^{7}x^{2}-5\cdot 10^{7}x+10^{8}\right|\\ R^{2}=0.992 \end{array}$$

where *RTT_Aver_*__1_*_Mbps_*(*ms*) is the RTT average value in ms, for 1 Mbps data transfer rate, and *x* is the distance between devices in cm.

### 6.2. Analytical Study for 2 Mbps Data Transfer Rate

**Figure 17.**
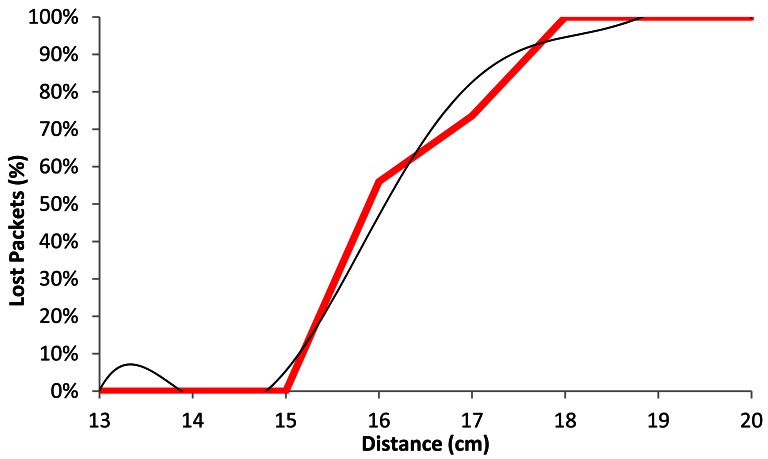
Average lost packets for 2 Mbps.

Figure 17 shows the percentage of lost packets as a function of distance for our real experiment (red line) and our model (black color), when the devices are using QPSK modulation with a data transfer rate of 2 Mbps. We see that for lower distances than 15.5 cm, the percentage of lost packets does not exceed 20%, while when the distance increases, the number of lost packets increases up to 70% (at around 17 cm). From approximately 18 cm, we obtain between 90% and 100% of lost packets.

In Figure 17, the black line follows polynomial expression of degree 6 (see Equation (5)). The correlation coefficient (*R^2^*) between both lines is 0.985:

(5) $$\begin{array}{c} \text{Lost}\_{\text{Packets}}_{2\text{Mbps}}\left(\%\right)=\left|-0.001x^{6}+0.102x^{5}-4.26x^{4}+93.84x^{3}-1.156x^{2}+7.550x-\;20.430\right|\\ R^{2}=0.985 \end{array}$$

where *Lost_Packets*_2_*_Mbps_*(%) is the percentage of lost packets for 2 Mbps data transfer rate and *x* represents the distance between wireless sensor nodes, in cm.

**Figure 18.**
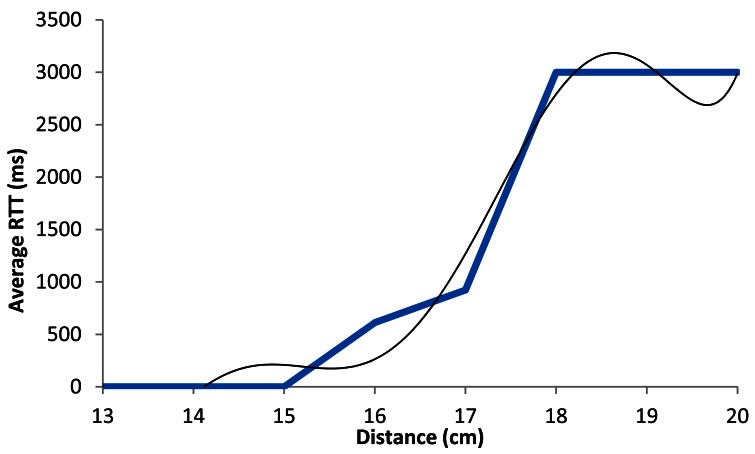
Average RTT for 2 Mbps.

Figure 18 shows the average RTT in ms as a function of the distance between devices, when the wireless sensor nodes are using QPSK modulation with a 2 Mbps data transfer rate. The blue line shows the average estimation of our measurements, while the black line shows our model. We see that in both cases, RTT values are below 300 ms for lower distances than 15.5 cm. When we increase the distance, the average RTT value increases up to 800 ms (at around 17 cm). But, from 17 cm, the graph increases very rapidly up to 3,000 ms (at 18 cm), where all packets are lost.

In Figure 18, the black line follows a polynomial expression of degree 6 (shown in Equation (6)), whose correlation coefficient (*R^2^*) with the real measurements is 0.976:

(6) $$\begin{array}{c} {\text{RTT}}_{\text{Aver}\_2\text{Mbps}}\left(\text{ms}\right)=\left|5.090x^{6}-501.4x^{5}+20.479x^{4}-44.374x^{3}+5\cdot 10^{6}x^{2}-\;3\cdot 10^{7}x+\;9\cdot 10^{8}\right|\\ R^{2}=0.976 \end{array}$$

where *RTT_Aver__*_2_*_Mbps_*(*ms*) is the average RTT value in ms, for 2 Mbps data transfer rate, and *x* represents the distance between devices, in cm.

### 6.3. Analytical Study for 5.5 Mbps Data Transfer Rate

**Figure 19.**
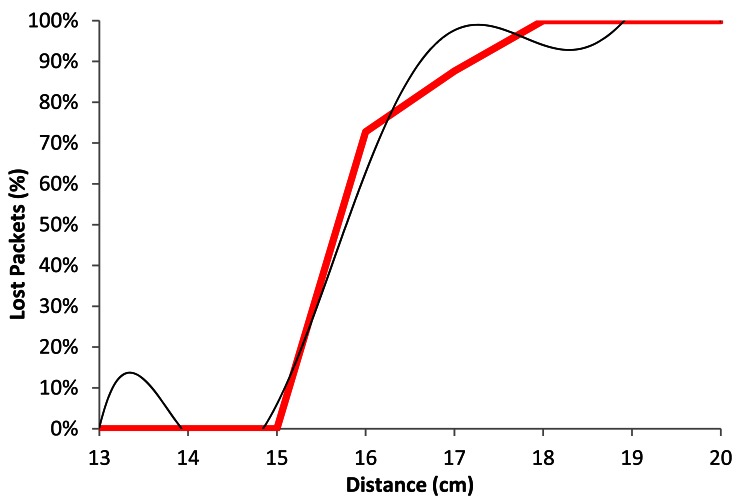
Average lost packets for 5.5 Mbps.

Figure 19 shows the average percentage of lost packets as a function of the distance between the wireless sensor nodes when both nodes are using CCK modulation with 5.5 Mbps data transmission rate. The red line shows the average of the gathered measurements, while the black line shows our model. In this case, the highest increase is obtained from 15 cm (below this value we always obtain very few lost packets, less than 10%) to 16 cm (where the lost packets percentage increases to around 70%). From 16 cm to 18 cm there is between 90 and 100% of lost packets.

The black line shown in Figure 19 follows a polynomial expression of degree 6. The correlation coefficient (*R^2^*) between both lines is 0.983. Equation (7) shows the black line expression:

(7) $$\begin{array}{c} \text{Lost}\_{\text{Packets}}_{5.5\text{Mbps}}\left(\%\right)=\left|-0.002x^{6}+0.208x^{5}-8.609x^{4}+188.4x^{3}-2.306x^{2}+14.981x-\;40.313\right|\\ R^{2}=0.983 \end{array}$$

where *Lost_Packets*_5.5_*_Mbps_*(%) is the percentage of lost packets for 5.5 Mbps data transfer rate and *x* is the distance in cm between wireless sensor nodes.

**Figure 20.**
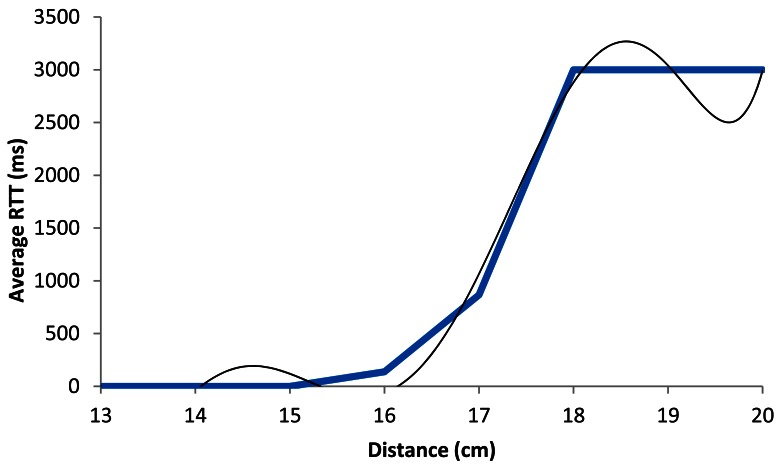
Average RTT for 5.5 Mbps.

Figure 20 shows the average RTT in ms as a function of the distance when the devices are using CCK modulation with 5.5 Mbps data transfer rate. The blue line shows the average of our measurements, while the black line shows our model. We observe that for smaller distances than 16.5 cm, the RTT value is always below 500 ms. But when we increase the distance between devices, the average RTT value increases up to 800 ms (at around 17 cm). From 17 cm, RTT value increases very quickly up to 3,000 ms at 18 cm. From that distance, all packets are lost.

The black line in Figure 20 has a polynomial expression of degree 6 (see Equation (8)). The estimate correlation coefficient (*R^2^*) between both lines is 0.992:

(8) $$\begin{array}{c} {\text{RTT}}_{\text{Aver}\_5.5\text{Mbps}}\left(\text{ms}\right)=\left|6.926x^{6}-681.2x^{5}+27.772x^{4}-60.071x^{3}+7\cdot 10^{7}x^{2}-\;5\cdot 10^{7}x+10^{8}\right|\\ R^{2}=0.992 \end{array}$$

where *RTT_Aver__*_5.5_*_Mbps_*(*ms*) is the average RTT value in ms, for 5.5 Mbps data transfer rates, and *x* is the distance between both wireless sensor nodes, in cm.

### 6.4. Analytical Study for 11 Mbps Data Transfer Rate

**Figure 21.**
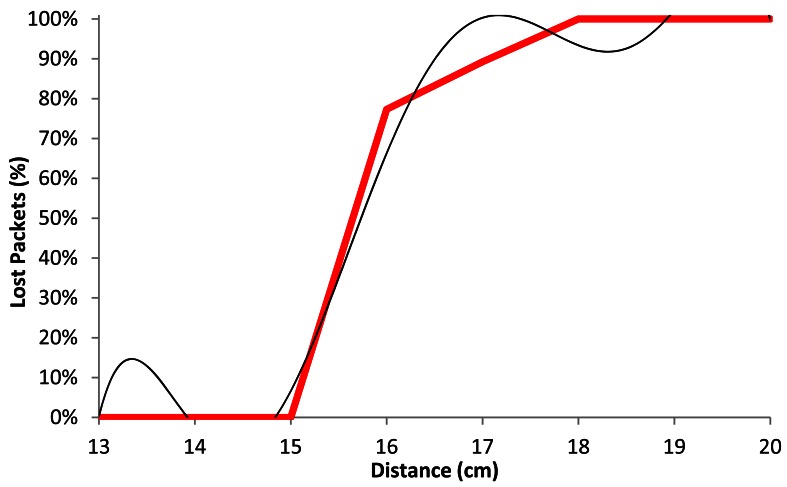
Average lost packets for 11 Mbps.

Figure 21 shows the average lost packets as a function of the distance between devices, when they are using CCK modulation with 11 Mbps data transfer rate. The red line shows the estimated average of our gathered measurements and the black line shows our model. We observe from 15 cm to 16 cm there is an increment from very few percentage of lost packets to approximately 80% of lost packets. From the 16 cm to 18 cm, the percentage of lost packets varies from 80% to 100%.

In Figure 21, the black line follows a degree 6 polynomial expression. It is shown in Equation (9). The correlation coefficient (*R^2^*) between both lines is 0.979:

(9) $$\begin{array}{c} \text{Lost}\_\text{Packets}s_{11\text{Mbps}}\left(\%\right)=\left|-0.002x^{6}+0.229x^{5}-9.468x^{4}+206.9x^{3}-2.531x^{2}+16.422x-44.150\right|\\ R^{2}=0.979 \end{array}$$

where *Lost_Packets*_11_*_Mbps_*(%) is the percentage of lost packets for 11 Mbps data transfer rates and *x* represents the distance between the wireless sensor nodes, in cm.

**Figure 22.**
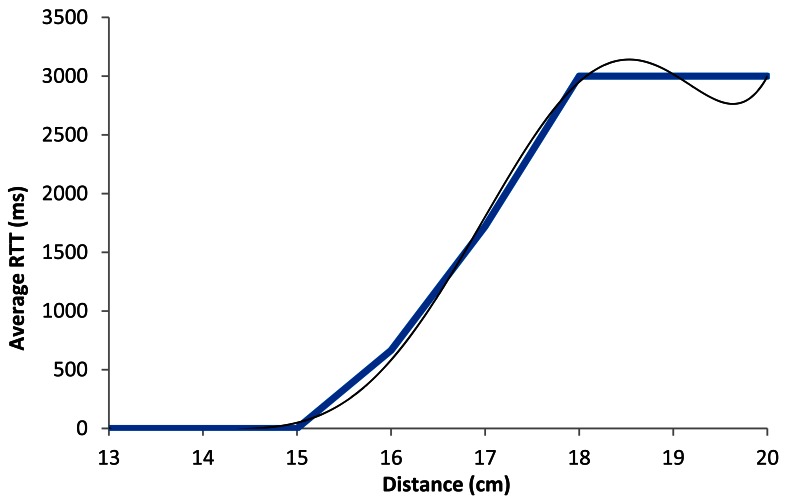
Average RTT for 11 Mbps.

Figure 22 shows the average RTT value in ms as a function of the distance between the wireless sensor nodes when they are using CCK modulation with 11 Mbps data transmission rate. The blue line shows the average of our measurements, while the black line shows our model. We observed a linear growth between 15 cm and 18 cm (although it is a little bit higher between 16 cm and 18 cm), where the average RTT value ranges from a few ms up to 3,000 ms.

The black line shown in Figure 22 follows a polynomial expression of degree 6. It is shown in Equation (10). The correlation coefficient (*R^2^*) between both lines is 0.998:

(10) $$\begin{array}{c} {\text{RTT}}_{\text{Aver}\_11\text{Mbps}}\left(\text{ms}\right)=\left|2.132x^{6}-205x^{5}+8.165x^{4}-17.236x^{3}+2\cdot 10^{6}x^{2}-1\cdot 10^{7}x+3\cdot 10^{7}\right|\\ R^{2}=0.998 \end{array}$$

where *RTT_Aver_*__11_*_Mbps_*(*ms*) is the average RTT value in ms, for 11 Mbps data transfer rates, and *x* is the distance between devices, in cm.

### 6.5. Analytical Model

On the other hand, we have modeled analytically the behavior (in terms of percentage of lost packets and average RTT) of the EM electromagnetic signals in freshwater when we transmit information between two wireless sensor nodes. Both the percentage of lost packets and the average RTT value, in ms, can be represented by a polynomial expression of degree 6. These equations let us predict the percentage of lost packets and the average RTT value that we will have in a real underwater wireless sensor network using our communication proposal.

The expression for the percentage of lost packets and the average RTT value follows the equation shown in Equation (11):

(11) $$Y=ax^{6}+bx^{5}+cx^{4}+dx^{3}+ex^{2}+fx+g$$

where the values of a, b, c, d, e, f and g variables depend on the distance and the data transfer rate. Table 4 shows the minimum and maximum values when Equation (11) is applied to the percentage of lost packets. Table 5 shows the minimum and maximum values when Equation (11) is applied to the average RTT. All other measured values of percentage of lost packets and average RTT fall within the ranges shown in Tables 4 and 5, respectively.

In both tables we see that the most different parameters are f and g. They are related with the growth of the linear part of the graphs and the point from which the graph grows rapidly. Moreover, we have also observed a great variation in the c, d and e parameters. They have relation with the curvature of the linear portion of these graphs and significantly less with the growth of the linear part of the graphic.

These equations allow us to determine the RTT values and the percentage of lost packets that can be obtained in an underwater communication system in similar conditions. Thus, they let us predict the behavior of an underwater sensor system.

## 7. Comparison with Other Communication Technologies

In this section, summarize the measurements provided by different published research about several underwater communication technologies in order to compare them with our proposal. We have classified them in three main types of communication technologies (acoustic waves, electromagnetic waves and optical waves). Table 6 shows this comparative. All works have been explained in the related work section (Section 2).

Our system achieved the highest data transfer rate. Second in line were the ones based on optical waves. But in these works on optical waves, the data rate improvement was achieved by increasing the power consumption of the devices, which should be avoided when working with wireless sensor nodes. The communication technology with the worst data transfer rate is the acoustic wave-based systems. However, these systems present the largest distances. We see that our proposal is among the tests with shortest distances. That is why we will focus our efforts on increasing these distances. Finally, we have observed that the published works on communications based on electromagnetic waves provide very little information on the type of tests performed. Thus, comparing the results provided by other researchers with our measurements, we found that our values of data transfer rates are pretty good, although the distances between nodes, are relatively small.

## 8. Conclusion

Research on underwater communications and the use of Underwater Wireless Sensor Networks is becoming a very hot topic because of the appearance of new marine/oceanographic applications. Communications based on EM wave transmission offer great benefits such as the increase of the data rate of the link to transmit more information.

In this paper, we have performed several tests at different frequencies and modulations, in order to check several parameters such as the minimum depth, distance between devices and signal transmission characteristics. These tests have been performed in the first seven channels that are specified in the IEEE 802.11 standard for the 2.4 GHz ISM frequency band (which is the frequency range between 2.412 GHz and 2.442 GHz).

After having gathered all these measurements, we highlight several issues. On one hand, we observe that the modulation (and thus the data transfer rates) with better performance are BPSK and QPSK. They have less than 30% of lost packets for distances shorter than 16 cm. There are also 30% of lost packets when QPSK modulation is used at 17 cm. Moreover, we observed that RTT values for 16 cm were around 25 ms when the wireless sensor nodes were working at 2.432 GHz. Thus, contrary to what we initially thought (the higher the frequency, the higher the attenuation), it seems that the communication system performance is improved slightly when it works at 2.432 GHz, compared with the results of the measurements obtained when it is working at 2.412 GHz.

We have observed that the increase of percentage of lost packets is higher from 15 cm to 16 cm than from 16 cm to 17 cm and from 17 cm to 18 cm. But when we measured the average RTT, there is a substantially greater increase from 17 cm to 18 cm than from 16 cm to 17 cm and from 15 cm to 16 cm.

Therefore, our underwater communication system has an optimum behavior at 16 cm, working at frequency of 2,432 GHz, with the BPSK and QPSK modulations. These modulations had also good performance at distances of 17 cm, working at 2.422 and 2.427 GHz, with a percentage of lost packets slightly above 30%.

Although our proposal provides short communication distances in underwater wireless sensor networks, we can use it for precision monitoring such as ecosystems contaminated by invasive plants (especially in ponds where there are some poisonous plants that can contaminate the water) or hazardous waste (e.g. in swamps, the quality of the water is different depending on the season because the water may contain some organic material that may be affected when it is warmer because the pH is different). In both cases the water cannot be used for human consumption, but, in some cases, it can be used by industries to run their plants and supply the water cooling system. Moreover, our proposal can be used to control the pollution of the water, which may come from industries and nearby roads, accurately.

Another application is for communicating with some parts of the neutrino telescope [38]. The neutrino telescope is an underwater structure located at the bottom of the Mediterranean Sea. Researchers are seeking ways to connect a hydrophone, for the positioning system of different parts of this structure. Until now, they have been using cables and penetrators, to unite the different parts. These pieces have a high economic cost. Using wireless communications, we would be reducing the cost of this material and would avoid the critical connections that can propagate a fault (or leak) through the system. Finally, the fact, that the distances between the devices are extremely small (practically in contact), means that the depth of this infrastructure is not a problem for wireless transmission of information. There are other applications such as, military applications, marine monitoring and even industrial applications such as marine fish farms [39], to reduce the deposition of organic waste on the seabed and to fight against environmental contamination

The proposed system provides several benefits. On one hand, it is cheap (because IEEE 802.11 devices are very cheap nowadays) and, on the other hand, it provides high data transfer rates, for the inclusion of all types of sensed data, even images.

We are also working on including more modulations and data bit rates in order to achieve large distances. Moreover, we have seen that the temperature of the water affects the distance, thus we will carry out more performance tests in future works. Finally we would like to design some specific antennae for underwater transmission, to transmit at 2.4 GHz.

## Figures and Tables

**Table 1.** Comparison of different wireless standards.

Standard	Frequency	Data Rate
IEEE 802.11b	2.4 GHz	11 Mbps
IEEE 802.11g	2.4 GHz	54 Mbps
IEEE 802.15.4	2.4 GHz	250 kbps
IEEE 802.15.4	868/915 MHz	40 kbps

**Table 2.** IEEE 802.11b/g modulations.

**Data Rates**	1 Mbps	2 Mbps	5.5 Mbps	11 Mbps

**Modulation**	BPSK	QPSK	CCK	CCK

**Table 3.** Data summary.

Distances	Modulation	1 Mbps	2 Mbps	5.5 Mbps	11 Mbps

		BPSK	QPSK	CCK	CCK
15 cm	Best frequencies (GHz)	All frequencies, have the same behavior			
	% of lost packets	0%	0%	0%	0%
	Average RRT (ms)	∼3	∼3	∼3	∼3

16 cm	Best frequencies (GHz)	**2.422** and **2.432**	**2.417, 2.422** and **2.432**	**2.412** and **2.417**	**2.417** and **2.422**
	% of lost packets	20% to 30%	10% to 20%	40% to 50%	10% to 20%
	Average RRT (ms)	28 and 20	18, 20 and 7	204 and 25	24, 208 and 547

17 cm	Best frequencies (GHz)	**2.427**	**2.422**	**2.427**	**2.427**
	% of lost packets	40%	30%	50%	70%
	Average RRT (ms)	28	46	3	17

18 cm	Best frequencies (GHz)	All frequencies, have the same behavior			
	% of lost packets	100%	100%	100%	100%
	Average RRT (ms)	∼3,000	∼3,000	∼3,000	∼3,000

**Table 4.** Min. and Max. values for percentage of lost packets.

	a	b	c	d	e	f	g
Min. value	−0.002	0.102	−0.9468	93.84	−2.531	8.817	−44.150
Max. value	−0.001	0.229	−4.260	206.9	−1.356	16.422	−20.430

**Table 5.** Min. and Max. values for average RTT.

	a	b	c	d	e	f	g
Min. value	2.132	−681.2	8.165	−60.071	2 × 10^6^	−5 × 10^7^	10^8^
Max. value	6.926	−205	27.772	−17.236	6 × 10^7^	−1 × 10^7^	9 × 10^8^

**Table 6.** Comparison table.

Reference	Technology	Working frequency	Length Wave	Modulation	Distance	Data transfer rates
[12]	ElectroMagnetic waves	3 KHz	N/app	N/av	40 m	100 bps
[13]	ElectroMagnetic waves	100 KHz	N/app	BPSK	6 m	1 Kbps
[14]	ElectroMagnetic waves	10 KHz	N/app	BPSK	16 m	1 Kbps
[15]	ElectroMagnetic waves	1 KHz	N/app	BPSK	2 m	1 Kbps
[16]	Optical Waves	N/av	420 nm	PPM	1.8 m	100 Kbps
[17]	Acoustic Waves	800 KHz	N/app	BPSK	1 m	80 Kbps
[18]	ElectroMagnetic waves	100 MHz	N/app	N/av	0.053 m	N/av
[31]	Acoustic Waves	12 KHz	N/app	MIMO-OFDM	N/av	24.36 Kbps
[32]	Acoustic Waves	24 KHz	N/app	QPSK	2500 m	30 Kbps
[33]	ElectroMagnetic waves	25 MHz	N/app	N/av	85 m	N/av
[34]	ElectroMagnetic waves	5 MHz	N/app	N/av	90 m	500 Kbps
[35]	Optical Waves	N/av	N/app	N/av	11 m	9.69 Mbps
[36]	Optical Waves	N/av	470 nm	N/av	10 m	10 Mbps
[37]	Acoustic Waves	70 KHz	N/app	ASK	70 m	0.2 Kbps
Our proposal	ElectroMagnetic waves	2.4 GHz (ISM Band)	N/app	BPSK	0.17 m	1 Mbps
Our proposal	ElectroMagnetic waves	2.4 GHz (ISM Band)	N/app	QPSK	0.17 m	2 Mbps
Our proposal	ElectroMagnetic waves	2.4 GHz (ISM Band)	N/app	CCK	0.16 m	5.5 Mbps
Our proposal	ElectroMagnetic waves	2.4 GHz(ISM Band)	N/app	CCK	0.16 m	11 Mbps

Note: N/app: Not applicable, N/av: Not available.

## References

[1] Akyildiz I.F., Pompili D., Melodia T. (2004). Challenges for efficient communication in underwater acoustic sensor networks. ACM Sigbed Rev..

[2] Sendra S., Lloret J., Garcia M., Toledo J.F. (2011). Power saving and energy optimization techniques for Wireless Sensor Networks. J. Commun. Acad. Publ..

[3] Garcia M., Sendra S., Atenas M., Lloret J. (2011). Underwater wireless *ad-hoc* networks: A survey. Mobile Ad hoc Networks: Current Status and Future Trends.

[4] Liu L., Zhou S., Cui J.-H. (2008). Prospects and problems of wireless communications for underwater sensor networks. Wirel. Commun. Mob. Comput.-Spec. Issue Underw. Sens. Netw..

[5] Stojanovic M. (1998). Underwater acoustic communication. Wiley Encyclopedia of Electrical and Electronics Engineering.

[6] Che X., Wells I., Dickers G., Kear P., Gong X. (2010). Re-evaluation of RF electromagnetic communication in underwater sensor networks. IEEE Commun. Mag..

[7] Chakraborty U., Tewary T., Chatterjee R.P. Exploiting the loss-frequency relationship using rf communication in underwater communication networks.

[8] Liebe H.J., Hufford G.A., Manabe T. (1991). A model for the complex permittivity of water at frequencies below 1 THz. Int. J. Infrared Millim. Waves.

[9] Somaraju R., Trumpf J. (2006). Frequency, temperature and salinity variation of the permittivity of seawater. IEEE Trans. Antennas Propag..

[10] (2007). IEEE 802.11 Standard: IEEE Standard for Information Technology—Telecommunications and Information Exchange between Systems—Local and Metropolitan Area Networks—Specific Requirements—Part 11: Wireless LAN Medium Access Control (MAC) and Physical Laye (PHY) Specifications.

[11] Martin F., Gorday P., Adams J., Leeuwen H.V. IEEE 802.15.4 PHY Capabilities. http://mentor.ieee.org/802.15/file/04/15-04-0227-04-004a-ieee-802-15-4-phy-layer-and-implementation.ppt.

[12] Chaitanya D.E., Sridevi C.V., Rao G.S.B. Path loss analysis of underwater communication systems.

[13] Sehgal A., Tumar I., Schonwalder J. Variability of available capacity due to the effects of depth and temperature in the underwater acoustic communication channel.

[14] Arnon S. (2010). Underwater optical wireless communication network. Opt. Eng..

[15] Wells I., Davies A., Che X., Kear P., Dickers G., Gong X., Rhodes M. Node pattern simulation of an undersea sensor network using RF electromagnetic communications.

[16] Frater M.R., Ryan M.J., Dunbar R.M. Electromagnetic communications within swarms of autonomous underwater vehicles.

[17] Anguita D., Brizzolara D., Parodi G. Optical communication for underwater wireless sensor networks: A VHDL-implementation of a physical layer 802.15.4 compatible.

[18] Nowsheen N., Benson C., Frater M. A high data-rate, software-defined underwater acoustic modem.

[19] Nowsheen N., Benson C., Frater M. Design of a high frequency FPGA acoustic modem for underwater communication.

[20] Jiang S., Georgakopoulos S. (2011). Electromagnetic wave propagation into fresh water. J. Electromagn. Anal. Appl..

[21] Sendra S., Lamparero J.V., Lloret J., Ardid M. Underwater communications in wireless sensor networks using WLAN at 2.4 Ghz.

[22] Balanis C.A. (1989). Advanced Engineering Electromagnetics.

[23] Chitode J.S. (2007–2008). Digital Communications.

[24] Andren C., Webster M. CCK modulation delivers 11 Mbps for high rate 802.11 extension.

[25] Raghunathan V., Schurgers C., Park S., Srivastava M.B. (2002). Energy-aware wireless microsensor networks. J. IEEE Signal Process. Mag..

[26] Vieira M.A.M., Coelho C.N., da Silva D.C., da Mata J.M. Survey on wireless sensor network devices.

[27] MatchPort Features http://www.lantronix.com/device-networking/embedded-device-servers/matchport.html.

[28] L52B LDO Datasheet http://www.farnell.com/datasheets/78785.pdf.

[29] Wang Z., Zeitoun A., Jamin S. (2003). Challenges and Lessons Learned in Measuring Path RTT for Proximity-Based Applications.

[30] Segal M. (2009). Improving lifetime of wireless sensor networks. Netw. Protoc. Algorithms.

[31] Li B., Zhou S., Stojanovic M., Freitag L., Huang J., Willett P. MIMO-OFDM over an underwater acoustic channel.

[32] Stojanovic M. Low complexity OFDM detector for underwater acoustic channels.

[33] Al-Shamma'a A.I., Shaw A., Saman S. Propagation of electromagnetic waves at MHz frequencies through seawater.

[34] Shaw A., Al-Shamma'a A.I., Wylie S.R., Toal D. Experimental investigations of electromagnetic wave propagation in seawater.

[35] Baiden G., Bissiri Y. High Bandwidth optical networking for underwater untethered telerobotic operation.

[36] Farr N., Chave A.D., Freitag L., Preisig J., White S.N., Yoerger D., Sonnichsen F. Optical modem technology for seafloor observatories.

[37] Won T.-H., Park S.-J. (2012). Design and implementation of an omni-directional underwater acoustic micro-modem based on a low-power micro-controller unit. Sensors.

[38] Ardid M. (2009). ANTARES: An underwater network of sensors for neutrino astronomy and deep-sea research. Ad. Hoc. Sens. Wirel. Netw..

[39] Garcia M., Sendra S., Lloret G., Lloret J. (2011). Monitoring and control sensor system for fish feeding in marine fish farms. IET Communications.

